# Lorenz’s classic ‘baby schema’: a useful biological concept?

**DOI:** 10.1098/rspb.2024.0570

**Published:** 2024-06-19

**Authors:** Yuri Kawaguchi, Bridget M. Waller

**Affiliations:** ^1^ Centre for Interdisciplinary Research on Social Interaction, School of Social Sciences, Nottingham Trent University, Nottingham NG1 4FQ, UK

**Keywords:** 'baby schema', cuteness, faces, attractiveness, Konrad Lorenz

## Abstract

Konrad Lorenz introduced the concept of a ‘baby schema’, suggesting that infants have specific physical features, such as a relatively large head, large eyes and protruding cheeks, which function as an innate releaser to promote caretaking motivation from perceivers. Over the years, a large body of research has been conducted on the baby schema. However, there are two critical problems underpinning the current literature. First, the term ‘baby schema’ lacks consistency among researchers. Some researchers use the term baby schema to refer to infant stimuli (often faces) in comparison with adults (*categorical usage*), while others use the term to refer to the extent that features contribute to cuteness perception (*spectrum usage*). Second, cross-species continuity of the ‘baby schema’ has been assumed despite few empirical demonstrations. The evolutionary and comparative relevance of the concept is, therefore, debatable, and we cannot exclude the possibility that extreme sensitivity to the baby schema is a uniquely human trait. This article critically reviews the state of the existing literature and evaluates the significance of the baby schema from an evolutionary perspective.

## 1. Introduction

Infancy is a critical period for an individual’s survival across species. It is estimated that at points in human history (e.g. 500 BC to AD 1950) approximately one-quarter of infants did not survive after their first birthday [1], so acquiring adequate care and protection from adults is vital. However, while it is optimal for offspring to demand maximum investment from their parents, it is optimal for parents to divide investment equitably among all offspring across their lifetime [2]. In the context of this parent–offspring conflict, infants evolve strategies to induce investment from parents, and likewise, parents glean information about the offspring to make investment decisions, using behavioural and physical cues. In the case of humans, the facial cues of infants seem to play an especially important role (e.g. [3]). Infant faces evoke unique neurological, physiological or psychological responses, e.g. by catching attention [4], activating brain areas involving reward processing and inducing impressions of ‘cuteness’ [5] and activating muscles associated with smiling [6,7]. Behavioural and neurological studies also suggest that individual variation in responses to infant stimuli can be associated with the quality of the rearing environment, including the quality of parenting behaviour (e.g. [4,8]). In sum, infant faces are salient stimuli and seem to serve an important role in the context of parental care in humans.

Individual differences in infant faces also affect caregivers’ behavioural outcomes. In humans, it is reported that mothers of more facially attractive infants are more likely to engage in affectionate and playful interaction with their infants while the mothers of less attractive infants tend to engage in routine caregiving [9]. Premature infants rated as more attractive by nurses thrive better compared with those rated less attractive [10]. Cuter infants are also perceived as healthier than less cute infants [11], so cuteness could act as a cue to potential health. The same argument could suggest that subtle cues in infant faces can have adverse effects. Indeed, infant faces with cues to low body weight [12], disorder [13] or premature birth [14] can be disfavoured, although this could be conditional upon the amount of available resources. An early study showed that physically abused children have facial proportions that look older than the age-matched non-abused children (although it is asserted that facial appearance is simply a specification for age level) [15]. Thus, infantile facial cues can affect parents’ important real-life decision-making about their investment. If infant faces have a unique role, what is it that makes them so special?

**Figure 1.**
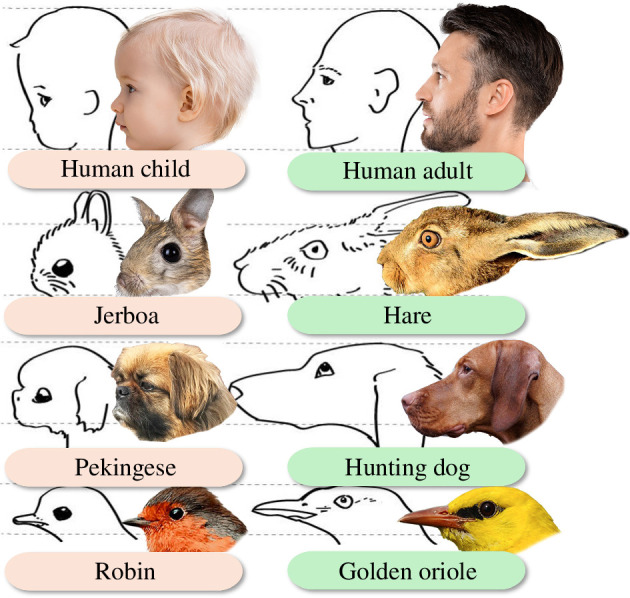
The original line drawing illustration of the baby schema (modified from [17], corresponding photographs added). The original illustration captures ‘the releasing schema for human parental care response. Left: head proportions perceived as ‘lovable’ (child, jerboa, Pekinese dog (sic) robin). Right: related heads that do not elicit the parental drive (man, hare, hound, golden oriole) [18, p. 155])’.

Konrad Lorenz and Nicolaas Tinbergen introduced the concept of ‘innate releasing mechanisms’, which was well established in the literature by the 1930s (e.g. [16]). According to this theory, animals respond to a certain type of stimulus with fixed action patterns. ‘Kindchenschema’ [17], which translates to ‘baby schema’, was originally proposed in relation to this idea and relates specifically to the mechanisms by which humans respond to small children. Namely, certain characteristics of children (or dolls or animals, figure 1) induce very specific affective consequences, urging the perceivers to perform instinctive parenting behaviours [17]. This emotional feeling is well accepted and often described as a feeling of cuteness but is difficult to pinpoint with a single word (at least in English). Lorenz originally used the words ‘niedlich (pretty)’, ‘süß (sweet)’ or ‘herzig (cute)’ for this feeling but is also expressed by others as ‘kawaii’ (Japanese, [19]), ‘kama muta’ (Sanskrit, [20]) or ‘aww’ (English, [21]).

However, since then the baby schema has begun to take on a life of its own, with a stronger emphasis on the specific visual characteristics of the stimulus, and now more than 100 scientific articles [22] and several review papers [13,23–27] have been published on the baby schema. For example, it has been demonstrated that the baby schema activates the reward system in the brain, and induces cuteness perception and caretaking motivation in humans [28,29]. This effect of infantile features on human cuteness perception is generalized to non-human animal faces [30,31] and even artificial objects such as cars [32], which attracts attention from applied fields of research such as product design (e.g. [33]). It may seem, therefore, that the baby schema hypothesis has been established on a solid body of empirical research. However, two critical points are missing. First, the term ‘baby schema’ lacks consistency among researchers which makes generalized conclusions difficult. Some researchers use the term baby schema simply to refer to infant faces or any infant stimuli (categorical usage), while others use the term to refer to specific features which contribute to cuteness perception (spectrum usage). Interestingly, Lorenz’s original paper [17] is also not always correctly cited. Second, empirical investigation of the baby schema has tended to be anthropocentric. While an evolutionary perspective is often taken in relation to the baby schema, cross-species data, which is needed to substantiate some of the key arguments, is conspicuously absent. In this article, we summarize how the term baby schema has been used in the literature to date, highlighting inconsistencies and reviewing existing findings relating to the baby schema in humans. We will then summarize existing findings in non-human animals and reflect on the implications for evolutionary continuity.

## 2. What is the ‘baby schema’?

### (a) Original argument by Lorenz

Lorenz published his original article mentioning the baby schema in German [17]. Although a brief summary of this discussion was also published later in English [18], the original argument is difficult to access for non-German researchers, which could be one of the reasons for confusion. Here, we discuss the original proposal about the baby schema and compare it with common interpretations by later researchers. An English translation of the original paper related to the baby schema (relevant parts) is provided in electronic supplementary material, appendix S1.

First, Lorenz described a set of characteristics that an object must have to trigger cuteness perception, as follows:

***‘1. Relatively thick, large head*** *, the optimal ratio of which to body size could perhaps be determined through experiments, as with Tinbergen's blackbirds. **2. The neurocranium, which is strongly predominant** in relation to the facial skull and protrudes with a domed forehead. **3. Eyes which are large and deep** in accordance with the aforementioned proportions, lying below the centre of the skull. **4. Relatively short, thick extremities** with large hands and feet. **5. Generally rounded body shapes**. **6. A very specific, soft and elastic surface texture**, corresponding to the fat layer of the healthy human child. **7. Round, protruding ‘chubby cheeks’**, without which the cuteness of the child’s head would be greatly reduced*.’ [17, p. 275], translated from German, emphasis added by the authors, see also electronic supplementary material, appendix S1).

Later, *‘clumsy movement’* was added to this [34].

In studies by later researchers, the baby schema is often described as ‘the large head, chubby cheeks, high and protruding forehead, large eyes, small nose and mouth’ [28,30,35–37]. However, one will notice that a small nose and mouth are not explicitly mentioned by Lorenz (although the nose and mouth, or muzzle or beak, are smaller in his illustration, figure 1). It is also worth noting that only a part of the characteristics of the baby schema are facial features. Non-facial characteristics are rarely mentioned in relation to the baby schema in later studies, but Lorenz included body shape, texture and even body movement as part of the baby schema.

Likewise, Lorenz’s illustration of the baby schema is well known and widely reproduced, but little attention has been paid to the caption. The caption states:

*‘The releasing schema for human parental care response. Left: head proportions perceived as ‘lovable’ (child, jerboa, Pekinese dog* (sic)*, robin). Right: related heads which do not elicit the parental drive (man, hare, hound, golden oriole)*’ [18, p. 155]).

The caption suggests, therefore, that the illustration serves to demonstrate characteristics of cute versus less cute and not young versus adult. With the exception of humans, each pair of images does not depict young versus adult animals within the same species, but instead compares closely related species/breeds of adult individuals on opposite spectrums of perceived cuteness (i.e. robin compared with golden oriol, jerboa compared with hare, Pekingese dog compared with hunting dog). It should also be noted that Lorenz did not assume that young animals are always cute. He argued that they are:

‘*Only ‘cute’ in proportion to the number and effectiveness of the characteristics mentioned’* [17, p. 275], translated from German, see also electronic supplementary material, appendix S1).

### (b) Inconsistent usage of the term among later literature

**Figure 2.**
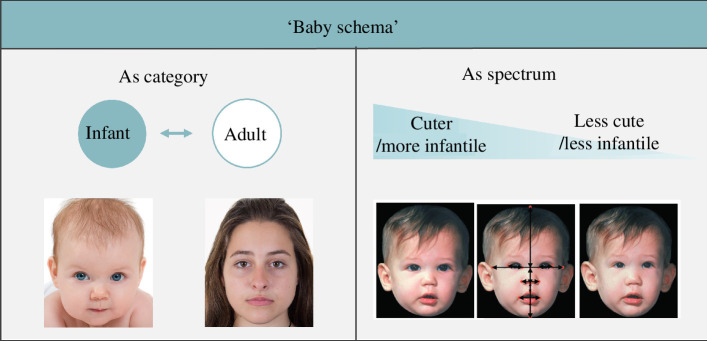
Examples of the stimuli based on the two main usages of the term ‘baby schema’. First, baby schema as a category referring to infant faces or infant stimuli in general. Second, baby schema as a continuous spectrum of characteristics contributing to cuteness perception or infantile face properties (image modified from Glocker *et al*. [36]).Examples of the stimuli based on the two main usages of the term ‘baby schema’.

In addition to the above seeming confusions over Lorenz’s original hypothesis and comments, the term ‘baby schema’ has been employed in various ways by different researchers. There are two primary uses (figure 2).

1. Baby schema as a category: some researchers use the term baby schema simply to refer to infant faces or any infant stimuli (versus adult faces or any adult stimuli). For example, Brosch *et al*. demonstrated that ‘baby schema’, which in that study means infant faces, captures attention in the dot-probe task [38]. In other studies, images of infants (both human and non-human) showing the face and some body parts were used as baby schema stimuli, which tend to be rated more positively than adult stimuli [7,39].
2. Baby schema as a spectrum: the more common usage of the term baby schema is as a continuous spectrum. The difference is that, unlike the studies above, these studies tend to refer to specific features that contribute to cuteness perception as the baby schema. Characteristics that receive the most attention are relatively large eyes, small nose and mouth, and protruding forehead. The baby schema is conceptualized as a matter of degree, and within this framework, it is possible that adult faces have a high baby schema. Certain facial parameters such as craniofacial profile, face width, forehead length, eye size, nose size and mouth size have been manipulated and the effect of the manipulation has been tested (e.g. [28,30,36]).

In summary, when the effect of the baby schema is tested, the stimuli can be infant faces/infant stimuli (as a category) or certain cute features (as a spectrum). This may be relevant to two different steps involved in perceiving infants in humans proposed by Hildebrandt & Fitzgerald [3]. That is, a positive reaction is elicited by the general appearance of an infant (‘babyishness’), while the robustness of the reaction is determined by the degree of individual attractiveness (‘cuteness’). The above-mentioned inconsistency of the terminology seems at least partly related to this confusion between ‘babyishness’ and ‘cuteness’. That is, all infants or infant faces (categorical usage of the baby schema) can be visually easily differentiated from adults or adult faces and evoke specific reactions including cuteness perception from perceivers; but the extent of the effect can be influenced by the degree of specific cute characteristics (spectrum usage of the baby schema). Whenever the ‘baby schema’ is discussed, therefore, it should be carefully considered whether it means general infant stimuli or a certain set of cute or infantile characteristics. Moreover, it should also be noted that the various outcomes induced by different types of ‘baby schema’ stimuli are often lumped as ‘baby schema effects (BSE)’, encompassing cuteness perception, preference and/or physiological responses (e.g. [32,39–41]).

## 3. Is there evidence for a ‘baby schema’ in humans?

### (a) Baby schema as a category

Humans can easily distinguish between conspecific infants and adults based on the face. Indeed, the mechanisms underlying the processing of adult and infant faces are somewhat independent in humans [42]. The ability to distinguish adult and infant faces also emerges very early in development, as even 9-month-old infants can categorize adult and infant faces [43]. Infant faces seem to evoke unique responses from perceivers in addition to being distinguished. First, infant faces affect gaze allocation. Brosch *et al*. investigated whether infant faces capture more attention than adult faces in a dot-probe task [38]. In this task, a pair of adult and infant face images were presented, followed by a target which the participants were required to respond to. Response times were shorter when the target was presented on the same side as the infant face (compared with the adult face) when presented in the right visual field. This attentional bias towards infant faces has also been reported in several other studies (for a review, see [4]) and appears to be influenced by racial [44] (but see also [45]) as well as species-specific [38] characteristics of the stimuli. The extent of attentional bias towards infant faces in mothers correlates with the quality of the interaction with their infants [4]. Attention bias for infant faces has also been demonstrated with a free-viewing eye-tracking task. Compared with males, female participants looked longer at infant faces than adult faces when paired together [46]. Such female bias is also reported in motivation to view infant face stimuli compared with adult faces [47,48] (but see [49] for contrasting evidence), where the participants could control the viewing time for different images. These gender differences are interpreted as reflecting historical sex differences in human parental care. A differential neural basis for processing infant and adult faces has also been shown by many studies (for a review see [26]). When responses to infant faces versus adult faces were examined using magnetoencephalography(MEG), e.g. specific brain activity was found to occur in the medial orbitofrontal cortex, which mediates reward processing [5]. Besides those visual features, auditory and olfactory features of infants are also suggested to induce a cuteness response [27].

### (b) Baby schema as a spectrum

Many human studies have investigated the effect of the baby schema in terms of how the extent of specific (often facial) infantile features contribute to cuteness perception, thereby considering the baby schema to be a spectrum. The original argument about the baby schema by Lorenz [17] was based on visual inspection without any quantitative or systematic observation. Thus, the exact facial properties that contribute to cuteness perception should, as a first step, be empirically investigated. Although some studies have focused on non-facial baby schema characteristics such as body shape proportion [50], the majority of studies have focused on faces. Interestingly, in contrast to the profile illustration by Lorenz, most studies have focused on frontal faces with some exceptions studying craniofacial profile shape with line drawing stimuli [23,51,52]. One study [53] asked people to rate the cuteness of unmanipulated infant face photographs of different ages and measured 14 facial features for each face. As a result, infant faces rated as cute were likely to have shorter and narrower features, large eyes and pupils, and a large forehead. Based on this study, Glocker *et al*. manipulated certain facial parameters including face width, forehead length, eye size, nose size, mouth size, to manipulate the degree of baby schema and asked the participants to rate cuteness and their motivation to take care of them [36]. High baby schema faces, which had wider faces with a larger forehead, bigger eyes, smaller nose and smaller mouth, were rated as cuter and induced more caretaking motivation than low baby schema or unmanipulated faces. This method is recognized as a standard way to manipulate facial baby schema and has since been used in other studies [8,28,30,54,55]. These studies confirmed that faces with such high baby schema properties trigger longer viewing times, as well as influence attractiveness ratings. In addition, there have been studies aimed at investigating neural responses to the degree of baby schema, but with mixed results. Enhanced brain activation of the nucleus accumbens (mediating reward processing) by high baby schema faces was reported in one study [28] while no differences in neural processing were found in two studies by another group [8,54]. One possible reason for this discrepancy (suggested by the authors) is that the former research group tested nulliparous young women (aged 20s) while the latter group tested mothers (aged 20s to 40s), suggesting that prior parenting experience may be an important factor. However, whether parental status impacts responses to the baby schema (in any regard) has yet to be demonstrated. Some research has shown that neural responses to infant stimuli can differ depending on parental status (e.g. [56]). Other studies, however, have reported that infant faces induce similar responses from parents and non-parents (e.g. [57]).

Although Glocker *et al*.’s method [36] to measure (or modify) the degree of the baby schema is frequently used and has also been applied to faces of non-human animals [30,35], there are some issues with this approach. These studies have focused on a predetermined set of independent facial features in a top-down manner. Consequently, the ecological relevance is unclear. Specifically, we still do not really know whether actual infant faces exhibit these features and whether these are all necessary and sufficient characteristics of the baby schema. Moreover, ‘a non-additive gestalt factor’ can influence perceptions of cuteness [53]; that is, the variation of each facial feature is not independent. For example, several studies have pointed out that eye size alone is poorly correlated with cuteness [29,53,58]. The holistic nature of facial characteristics is overlooked when each facial part is independently measured. Some studies address these issues by manipulating facial features affecting cuteness perception in data-driven ways. For example, by using a landmark-based approach, the facial features of infants contributing to cuteness perception were identified specifically [58,59]. Consequently, one study found that infant faces that were rated as cuter had a wider forehead, a smaller chin, a big smile and round chubby features [58]. Other studies manipulated the cuteness of infants (and adults) by the transformation between more and less cute face prototypes, which are determined based on cuteness ratings [60–62].

### (c) Baby schema beyond human faces

The original idea of the baby schema proposed that human cuteness perception is not limited to ones’ own species but is instead invoked when humans view other animals too. Therefore, several studies have investigated human cuteness perception towards non-human animal stimuli. Kruger tested impressions of the unmanipulated images of young animals of different species (birds and reptiles) from both semi-precocial species (i.e. requiring parental care) and super-precocial species (i.e. not requiring parental care) [63]. He found that people perceive infants of semi-precocial species as more neotenous than super-precocial species, and people are more motivated to interact with infants of semi-precocial species. This finding is argued as empirical evidence of the convergent evolution of neotenous features eliciting caregiving responses. Nevertheless, this study only investigated the perception of humans but not of the species. Thus, we cannot conclude whether these neotenous features have evolved as signals to advertise needs for nurturing care to the conspecifics, as we will discuss later. As far as we know, there is no study exploring whether facial characteristics impact behavioural outcomes (e.g. abandonment or investment) in non-human animals. Several studies have also investigated how different degrees of the facial baby schema characteristics in animals affect human cuteness perception. Borgi *et al*. [30] manipulated a set of facial features of dogs and cats, as well as humans, based on Glocker’s method [36] and studied gaze allocation in children. They found that children looked longer at high baby schema images compared with low baby schema images independent of species. Moreover, the degree of baby schema was measured for faces of Barbary macaques (based on this same measurement) and examined in relation to the first impressions by humans [35]. As a result, contrary to the authors’ predictions, baby schema was not correlated with perceived age or cuteness, but positively correlated with perceived health and femaleness. Additionally, one study transformed faces based on the difference between human baby and human adult faces and made more infant-like and adult-like versions of babies, adult humans and adult cats [31]. It is important to note that babyness and cuteness may not always be the same [37,64], however, infant-like manipulations in this study increased cuteness ratings similarly in all face types. This evidence suggests that humans use a common facial feature to code cuteness in human and animal faces. Another study also examined whether human cuteness perception of different species involves the same mechanism by using a visual adaptation paradigm [65]. In this study, after exposure to cute human infant faces, subsequent human infant faces were perceived as less cute and *vice versa* (after-effect). Importantly, exposure to cute or less cute puppy faces similarly affected subsequent cuteness judgement for human infant faces. Based on the result, the authors argued that there is a common mechanism that codes the cuteness of human and non-human infant faces.

The combined results from these studies suggest that the effect of the baby schema is, to some extent context independent, in the sense that it is not limited to conspecific faces. However, it is important to note that animal faces rated as cute by human perceivers may not hold a similar meaning for the animal conspecific. Indeed, results from research manipulating animal faces may not accurately reflect the infantile facial characteristics of the species since they were based on predetermined features of human facial cuteness. For example, in one study [30], the degree of the baby schema is manipulated in adult and infant faces of humans, dogs and cats based on Glocker’s method [36], and as a consequence, high baby schema faces in all species have several characteristics including bigger eyes. However, based on their measurement of facial parameters in original faces (20 images for each category), relative eye width (eye width divided by face width) was reported to be bigger in human infants (mean ± s.d.: 0.19 ± 0.01) compared with human adults (0.17 ± 0.01), while it is smaller in kittens (0.15 ± 0.01) compared with adult cats (0.16 ± 0.01) and there were no differences between adult dogs (0.12 ± 0.03) and puppies (0.12 ± 0.02). Thus, it may not always be the case that relatively large eyes or other facial characteristics, known as baby schema characteristics, are universally observed in infants across species. Even if a certain type of facial feature is shared in animal infants across species, they may not always manifest in an identical way to humans. This may not always pose an issue if the goal is to investigate the human perception of animal faces in the context of potential animal–human interactions, but it is important to take ecological validity into account within the context of conspecific interactions (or indeed in real-life animal–human interactions).

### (d) Are responses to the baby schema innate?

Another key aspect of the baby schema is that it is proposed to act as an ‘innate’ releaser of caretaking. Consequently, researchers have investigated the developmental aspect. There is evidence that not only adults but also children, despite being too immature to take care of infants, exhibit a preference for the baby schema. In one study, face images of animals, including humans, chimpanzees, rabbits, dogs and cats at different developmental stages, were presented to adult and preschool children [66]. The researchers found that children’s judgement of cuteness closely corresponded to that of the adults. Manipulation of the facial baby schema has been shown to affect the cuteness rating and gaze allocations of children aged from 3 to 6 years [30]. This early preference for infantile faces aligns with the idea that baby schema serves as an innate releasing mechanism for parenting behaviour [17]. However, it is possible that pre-school children are already influenced by their socio-cultural environment. Indeed, when examining the development of preference for infant stimuli (a categorical aspect of the baby schema), contrasting results emerged. Different timings have also been reported regarding the emergence of a preference for infants. For example, one study reported that children aged 7–10 years old already prefer infants over adults [67], while the other reported that the preference for infants only became apparent at grade 8 (aged 13–14 years old) for girls and only 2 years later for boys [68]. It is also unclear when the looking bias for infants emerges. When pairs of adult and infant faces were presented, 3.5- and 6-month-old infants looked at adults longer than at infants [69]. In summary, it remains uncertain whether the human response to the baby schema, in any regard, develops over time or is relatively fixed.

## 4. Is there a ‘baby schema’ in non-human animals?

### (a) Assumptions of cross-species universality of the baby schema

It is widely believed that the psychological/behavioural outcomes of the baby schema are universal across mammalian species where caretaking is important (e.g. [70]). Lorenz wrote as follows:

*‘As with all innate schemas in humans and probably also in animals, where it is naturally impossible to say with any certainty, the response to the schema of childlike characteristics is also associated with very specific, autonomous and incomparable feelings and affects, with a very specific experience…*’ [17, p. 274], translated from German (see also electronic supplementary material, appendix S1).

Contrary to the broadly spread view, Lorenz did, therefore, not argue that there is a baby schema in non-human animals, but he did suggest that this might be possible. Nevertheless, the concept is heavily embedded within an evolutionary framework. First, some characteristic features of the baby schema assumingly reflect biological constraints. For example, the eyes and brain (which are accommodated in the forehead) develop earlier than other parts of the face, resulting in relatively larger eyes and a protruding forehead in infants [71]. Consequently, it is reasonable to expect that some features of the physical baby schema are also observed in other animals. Second, it assumes that it is adaptive for infants (and to some extent also adults) if adults’ caretaking motivation is triggered by certain characteristics of infants. To understand the evolutionary pathway of the baby schema, it is important to compare humans and non-human animals not as stimuli but as perceivers. For example, Kruger found that animal infants of species requiring parental care are perceived as more neotenous (cute, immature and helpless) and the author interpreted the results as supporting evidence of convergent evolution for infant paedomorphic features across non-human species. However, the target species were not domesticated animals (e.g. gull, penguin, crocodile and lizard), so it is unlikely that those features have evolved to induce caretaking motivation from humans. On the other hand, in companion animals, there is indirect evidence suggesting that having more pronounced baby schema features motivates humans to take care of them, thus proving adaptive. Dogs (*Canis familiaris*), who exhibit more paedomorphic facial expressions (i.e. ‘puppy dog eyes’) spend shorter periods in a shelter before being re-homed [72]. Another example is that the attractiveness of dog puppies to humans peaks at the weaning age, when they are most likely to get benefit from human adaptation [73]. These findings suggest that having infantile (cute) characteristics likely favours dogs. However, the adaptive value of having such characteristics in non-companion animals remains unclear. Thus, the critical question is whether having specific infantile characteristics in non-human animals (in general) induces more parental care and related responses from the same species. While research on the baby schema has largely focused on humans as the perceiving observers, we will review relevant studies tested in animals, focusing on non-human primates.

### (b) Baby schema as a category

There is evidence that animals are attracted to visual stimuli of infants of their own species. Some studies have investigated their motivation to view infant stimuli. Pryce *et al*. investigated if viewing conspecific infant stimuli served as a reward in common marmosets (*Callithrix jacchus*) [74]. They found that marmosets learned an operant behaviour (i.e. pressing a lever) to get visual access to an infant replica cast from 2-day-old infants, suggesting that there is motivation to view the visual stimuli of infants. A rewarding effect of infant visual stimuli had also previously been reported in infant rhesus macaques (*Macaca mulatta*) [75], but not in chimpanzees (*Pan troglodytes*) [76] by using a similar sensory reinforcement task. Other studies employed a free-viewing task with conspecific visual stimuli and reported longer looking duration for infants compared with adults in non-human primates, including Campbell’s monkeys (*Cercopithecus campbelli*) and Japanese macaques (*Macaca fuscata*) [77], Barbary macaques (*Macaca sylvanus*) [78] and chimpanzees but not bonobos (*Pan paniscus*) [79]. Nevertheless, when tested with a touch panel task requiring spatial orientation to the target such as a dot-probe test, the attentional bias for infant faces was not observed in either Japanese macaques [80] or chimpanzees [81] in contrast to the abundant evidence in humans (for a review see [4]). These comparative studies suggest that at least some primate species spontaneously pay more attention to infants in a free-viewing task, and they are motivated to perceive infant stimuli, but there is mixed evidence.

### (c) Baby schema as a spectrum and other infantile features

Some characteristic features of the baby schema arise at least partially from biological constraints such as the differential timing of the development of facial parts [71]. Consequently, it is reasonable to assume that some features of the physical baby schema are also observed in other animals. Infantile facial characteristics (but not necessarily ‘cute’ characteristics) have been systematically measured in infants of five great ape species including humans, chimpanzees, bonobos, mountain gorillas (*Gorilla beringei beringei*) and Bornean orangutans (*Pongo pygmaeus*) using landmark-based geometric morphometrics [82]. The authors consistently observed the following characteristics in infants across species: (i) relatively bigger eyes located lower in the face, (ii) a rounder and vertically shorter face shape and (iii) an inverted triangular face shape. These characteristics largely overlapped with the baby schema features listed by Lorenz. Additionally, the researchers identified facial features unique to infants of specific species. It is possible that the baby schema characteristics may produce similar effects in both humans and other altricial animals (at least some species), given the significant role faces play in communication in non-human primates [83] and the evolutionary advantage of attraction to infants for their survival. Human preference for objects with curved contours, which may have some relevance to the baby schema [84,85], is also shared with non-human primates [86]. However, as far as we know, there is no comparative research specifically testing the effect of the baby schema in terms of a certain morphological set of characteristics, which typically elicit cuteness perception in humans.

Scientists have investigated the effect of other visual cues of infants in non-human primates, e.g. infantile colouration or natal coats. Many primate species exhibit distinct coat or skin colouration during infancy, which differs from that of adults. While the evolutionary functions of infantile colouration remain a topic of debate, one influential view is that it serves a potential function for inducing alloparenting behaviour [87,88]. Consequently, the function of such colouration may overlap with that of the baby schema. Several studies have attempted to examine the mechanical function of this colouration. Two studies investigated female rhesus macaques' gaze duration and approach behaviour to infant stimuli with different colouration. The preference for neonatal facial colouration was found in one study [89] but not in a subsequent one [90]. The different results are probably owing to methodological differences (i.e. real animals dyed in different colours versus colour-manipulated images used as the stimuli). The effect of infantile face colouration in chimpanzees has also been tested by eye-tracking experiments [79]. This study found that chimpanzees looked at infant faces longer than at adult faces, but the looking time for infant faces decreased significantly when infantile colouration was removed. In another study [91], facial cues to age categorization were tested in chimpanzees. They systematically manipulated facial shape and colour, ranging from the average infant face to the average adult face. The results indicated that facial colour uniquely expressed by infants played a more important role in age categorization in chimpanzees than facial shape. These studies examining the role of infantile colouration partially suggest that, in some primate species, such colouration may serve a function similar to the baby schema in humans.

In summary, these studies suggest that infant stimuli attract visual attention in non-human primate species, and there is increased motivation to observe them in some species. However, the effect of the baby schema in regard to specific facial characteristics, which are specifically interpreted as cute, has not been tested. Consequently, cues that trigger responses to infants in non-human primates may not always be owing to facial morphology like in humans, but could instead be attributable to other characteristics such as infantile colouration. It remains unclear if the specific morphological characteristics of infant faces just reflect physical constraints or specifically have evolved as a signal of nurturing needs across species. In other words, we do not know whether or not infantile characteristics have evolved for the purpose of inducing parenting. To disentangle these, future studies are needed to examine whether specific evolutionary pressures have favoured infants with more baby schema features across non-human primate species. Human infants are especially altricial and require extensive care from both related and unrelated adults, and humans have extensive cooperative breeding systems for rearing them. Thus, it is possible that the high sensitivity to and preference for infants may have been highly adaptive and selected especially in humans.

## 5. Conclusion

First, we reviewed the different uses of the term ‘baby schema’, considering how it can be conceptualized as both a category and a spectrum. Although both could be relevant in different contexts, it is crucial to be precise in terminology to facilitate communication, especially across different fields and interdisciplinary research. Sometimes, using the term ‘infant face’ may be more accurate than ‘baby schema’ likewise ‘attentional bias’ or ‘preference’ may be more suitable operational terms than ‘baby schema effect’. For example, stating ‘we investigated if infant face images are rated as more attractive than adult face images’ is much more informative than stating ‘we investigated the baby schema effect’. It is also important to avoid assumptions about what has been demonstrated empirically, and what has not. Next, we reviewed the existing human studies on infantile facial characteristics and perceptions of cuteness. Several studies have emphasized the applicability of facial properties affecting cuteness perception by humans to other animals’ faces. However, there are mixed results regarding the innate nature of preference for the baby schema. Lastly, we explored relevant studies in non-human primates to gain insight into the evolutionary origin of the baby schema. The effect of the baby schema, specifically in terms of facial characteristics, has not been systematically verified across species. Therefore, there is a need for more cross-species studies. This is because high sensitivity to facial baby schema could be a uniquely human trait since human infants especially require extensive care from adults. To understand this, the key question is whether having immature characteristics is associated with receiving more parenting/alloparenting both at the individual and species level. More specifically, expressing more infantile features than other individuals could affect the behaviour of the group members surrounding the individual and give advantages to the holder. Also, if the baby schema does act as a releaser of caretaking, it should be expressed in a more reliable and explicit manner, especially in the species that show higher level of parenting. The review should serve as an important step in revisiting the original argument about the baby schema and a call to fully integrate an evolutionary perspective into this field of research.

## Data Availability

Supplementary material is available online [92].
